# Mechanical stress on the rod in posterior spinal fusion with and without rod fracture: a finite element analysis

**DOI:** 10.1186/s12891-025-09377-2

**Published:** 2025-11-28

**Authors:** Tomohisa Inoue, Yoh Kumano, Jun Sugaya, Ken Okazaki, Toru Doi

**Affiliations:** 1 Department of Orthopaedic Surgery, Tokyo Women’s Medical University, 8-1 Kawada-cho, Shinjuku-ku, Tokyo, Japan; 2 Department of Spine Surgery, Tokyo Yamate Medical Center, Japan Community Healthcare Organization, 3-22-1 Hyakunin-cho, Shinjuku-ku, Tokyo, Japan

**Keywords:** Rod fracture, Posterior spinal fusion, Finite element analysis, Mechanical stress

## Abstract

**Background:**

Rod fracture (RF) is a common complication of posterior spinal fusion surgery, with a high reoperation rate. This study evaluated the mechanical stress on the rods and intervertebral discs under various loading conditions in a simplified short-segment lumbar fusion model (L3-S1) with and without a unilateral rod fracture (URF) via 3D-computed tomography finite element analysis (CT/FEA), to clarify the spinal motion directions most associated with the mechanical stress on the rods.

**Methods:**

Two 3D nonlinear finite element models of L3–S1 posterior spinal fusion were created using the preoperative CT images of 10 patients: (1) a no rod fracture (NRF) model and (2) a URF model with a simulated left RF at L4/5. A 10-Nm load was applied in six directions (flexion, extension, right and left lateral bending, and right and left axial rotation), and the maximum von Mises stress on the right rod at L4/5 and the minimum principal stress on the L2/3 adjacent and L4/5 intervertebral discs were measured and compared between both models. Furthermore, the maximum von Mises stress on the right rod was also compared among six loading directions.

**Results:**

Mechanical stress on the L4/5 intervertebral disc was significantly higher in the URF model under all loading directions, but there was no significant difference in the mechanical stress on the adjacent L2/3 intervertebral disc between both models. In the NRF model, mechanical stress on the right rod was significantly higher under left and right axial rotational loadings versus the other loading directions. In the URF model, mechanical stress on the right rod was significantly higher under left lateral bending and axial rotational loading versus flexion loading.

**Conclusions:**

After posterior spinal fusion surgery, restricting spinal axial rotation and lateral bending toward the fractured side may help reduce mechanical stress on the rods.

## Introduction

Adult spinal deformity is a common spinal disorder characterized by spinal deformity and malalignment, resulting in chronic low back pain, impaired mobility, and reduced quality of life [1]. Its reported incidence ranges from 1.4% to 32% [2–4], and spinal instrumentation surgery has become more common for severe cases in recent years [5]. Although favorable clinical results after surgery have been reported [6], various postoperative complications, such as instrumentation failure, can also occur [7]. Rod fracture (RF) is one of the most common postoperative complications after spinal fusion surgery, with a relatively high incidence ranging from 6.8% to 38.8% [8–10]. RF can cause spinal instability and nonunion at the level of RF, often requiring revision surgery in 41.2% to 87% of cases [9, 11]. Notably, the reoperation rate is significantly higher for bilateral RF (75%–91%) compared to unilateral rod fracture (URF, 21%–43%) [8, 11]. Therefore, effective strategies for preventing RF are essential to reduce the risk of reoperation and improve postoperative clinical outcomes.

The risk factors for RF include obesity, osteoporosis, smoking, preoperative sagittal imbalance, and postoperative malalignment [8, 11–16]. To prevent RF, the use of stiffer constructs (i.e., using more rigid or multiple rods) have been recommended [17]. Previous studies using 3D-computed tomography finite element analysis (CT/FEA) have investigated the mechanical stress on rods to determine the optimal rod materials, length, and accessory rod placement [18, 19]. However, due to a scarcity of data on how mechanical stress changes under various loading directions, the optimal spinal motion restrictions to prevent RF and its progression remain unclear.

Accordingly, this study evaluated the mechanical stress on rods and intervertebral discs under various loading directions in no rod fracture (NRF) and URF models via CT/FEA. This can be valuable for determining the most appropriate postoperative management for preventing RF and its progression.

## Methods

### Study patients

Preoperative CT images from 10 patients who underwent posterior lumbar decompression at our institution between January 2023 and December 2024 were used to construct 3D spinal models. Written informed consent was obtained from all participants. To investigate the effects of loading direction and URF on rod and intervertebral disc stress and eliminate confounding factors (e.g., sagittal imbalance, postoperative malalignment, and osteoporosis), the following exclusion criteria were applied: severe lumbar degenerative changes, kyphoscoliosis requiring spinal corrective fusion surgery, and age ≥ 65 years who were associated with low bone mineral density. Patient demographics, including age, sex, body mass index (BMI), and diagnosis were also examined.

### 3D Spinal CT/FEA Model Creation and Mechanical Stress Measurement

**Fig. 1:**
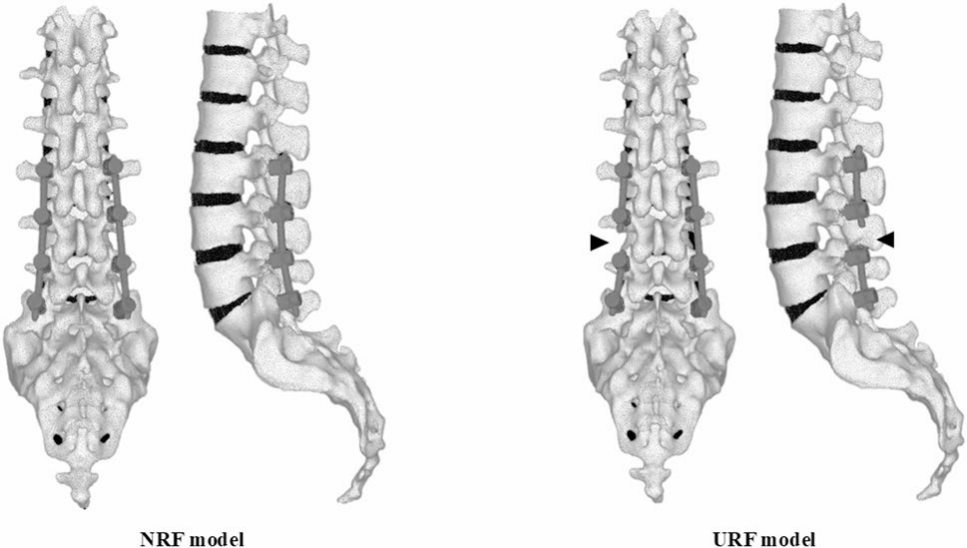
Posterior and lateral views of the NRF (left) and URF (right) models. Arrow head indicates RF site

Preoperative CT images from thoracic spine to the pelvis were acquired using a calibration phantom (QRM-BDC; QRM, Germany). DICOM data was imported into the CT/FEA software (Mechanical Finder version 13.0 software, RCCM, Tokyo, Japan) to construct 3D nonlinear finite element models from T12 to the sacrum. A finite element model from T12 to the sacrum was equipped with triangular shell elements (thickness, 2 mm; size, 3 mm) for the outer surface of the cortical bone and tetrahedral solid elements with a size of 2 mm, which were determined based on previous studies [20, 21]. The material properties of the vertebrae, intervertebral discs, spinal ligaments, and spinal instrumentation were adopted from prior studies [22–25], (Table 1). Mechanical Finder has been widely used and validated in previous biomechanical studies of the spine [18, 20, 21], ensuring the validity of finite element modeling approach in this study. Pedicle screws (diameter: 6.5 mm, length: 40 mm) and rods (diameter: 5 mm, titanium alloy) were modeled using Metasequoia^Ⓡ^ (version 4, Tetraface, Tokyo, Japan). To eliminate the confounding factors such as spinal alignment and deformity severity, and to focus specifically on the influence of loading direction and RF on the mechanical stress changes of rods or intervertebral discs, a simplified short-segment posterior spinal fusion model from L3 to S1 was adopted. Pedicle screws were placed from L3 to S1, and rods were attached accordingly. Regarding the contact condition between the pedicle screws and the vertebrae, no frictional contact elements were defined; therefore, the screw–bone interfaces were assumed to be perfectly bonded. Two models were developed: (1) an NRF model with bilaterally intact rods, and (2) a URF model in which rod fracture was not modeled as a dynamic fracture process but rather was simulated by removing the left rod between the L4 and L5 pedicle screws (Fig. 1). The RF site was set at L4/5 level because L4/5 was the most mobile lumbar segment [26] and is considered the most susceptible to the occurrence of RF within the L3-S1 fusion model. The sacroiliac joint surface was restrained, and a 10-Nm load was applied in 6 directions (flexion, extension, right/left lateral bending, and right/left axial rotation) using a cross-shaped 3D block at the superior endplate of T12. The number of load steps was set to 50 based on a load-step convergence analysis, which confirmed that this condition provided stable stress results. The minimum principal stress (megapascal, MPa) on the intervertebral discs at the adjacent level (L2/3) and RF level (L4/5), as well as the maximum von Mises stress (MPa) on the right rod at L4/5 were measured under each loading direction for each model. Since principal stress indicates tensile stress as a positive value and compressive stress as a negative value, the minimum principal stress was used to evaluate the compressive stress on the intervertebral discs. The absolute value of the minimum principal stress was used for statistical analysis to compare the magnitude of the compressive forces on the intervertebral discs. Meanwhile, the mechanical stress on the rods was represented by the maximum von Mises stress as an equivalent stress. Contours of maximum von Mises stress on the rods under flexion and right axial rotational loading in the NRF and URF models were generated.

**Table 1:** Material properties

	Elastic modulus (MPa)	Poisson’s ratio	References
Vertebrae	Inhomogeneous material from Keyak’s equation	0.4	[21]
Intervertebral disc	Hyperelastic, Mooney-Rivlin c1 = 0.1, c2 = 0.1		[20]
Ligaments			
Anterior longitudinal ligament	7.8 (ε < 12), 20 (ε > 12)	0.3	[22]
Posterior longitudinal ligament	10 (ε < 11), 20 (ε > 11)	0.3	[22]
Ligamentum flavum	15 (ε < 6.2), 19.5 (ε > 6.2)	0.3	[22]
Supraspinous ligament	8 (ε < 20), 15 (ε > 20)	0.3	[22]
Interspinous ligament	10 (ε < 14), 11.6 (ε > 14)	0.3	[22]
Transverse ligament	10 (ε < 18), 58.7 (ε > 18)	0.3	[22]
Capsular ligament	7.5 (ε < 25), 32.9 (ε > 25)	0.3	[22]
Implant (Titanium alloy)	110,000	0.34	[23]

*MPa* megapascal

### Statistical analysis

Descriptive statistics were reported as means (standard deviation, SD) for demographic data and median (interquartile range, IQR) for the measurements of minimum principal stress and the maximum von Mises stress, respectively. The minimum principal stress at the L2/3 and L4/5 intervertebral discs and the maximum von Mises stress on the right rods at L4/5 under each loading direction were compared between both models using a Wilcoxon signed-rank test. The Friedman test with Bonferroni correction was used for multiple comparisons of the maximum von Mises stress on the right rod among the six loading directions. All data analyses were performed using IBM SPSS, Version 29 (IBM Corp., Armonk, NY), with *P* < 0.05 indicating statistical significance.

## Results

### Patient demographics

The characteristics of 10 patients are presented in Table 2. The study population included 6 males and 4 females with a mean age of 42.4 ± 11.8 years, and BMI of 21.1 ± 2.9. All patients had a diagnosis of lumbar disc herniation.

**Table 2:** Demographic data of 10 patients

*n* = 10	
Age, years	42.4 ± 11.8
Sex, male/female	6/4
BMI, kg/m^2^	21.1 ± 2.9
Diagnosis, n	Lumbar disc herniation, 10

Values are reported as mean (standard deviation). *BMI* body mass index

### Mechanical stress on intervertebral discs

**Fig. 2:**
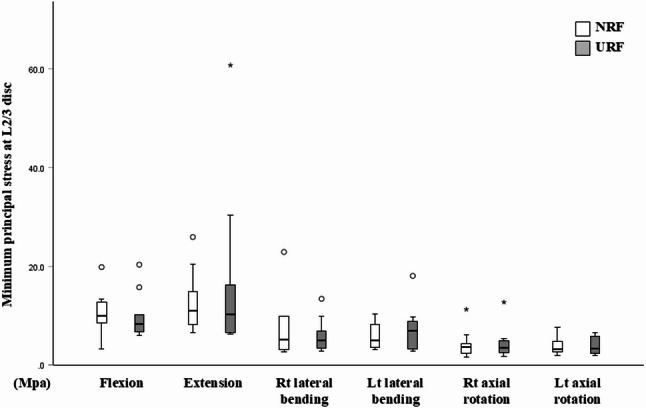
Minimum principal stress at L2/3 disc under 6 loading directions in the NRF (white box) and URF (grey box) models. A circle represents a mild outlier (more than 1.5 times the interquartile range beyond the box), while an asterisk represents an extreme outlier (more than 3 times the interquartile range beyond the box)

**Fig. 3:**
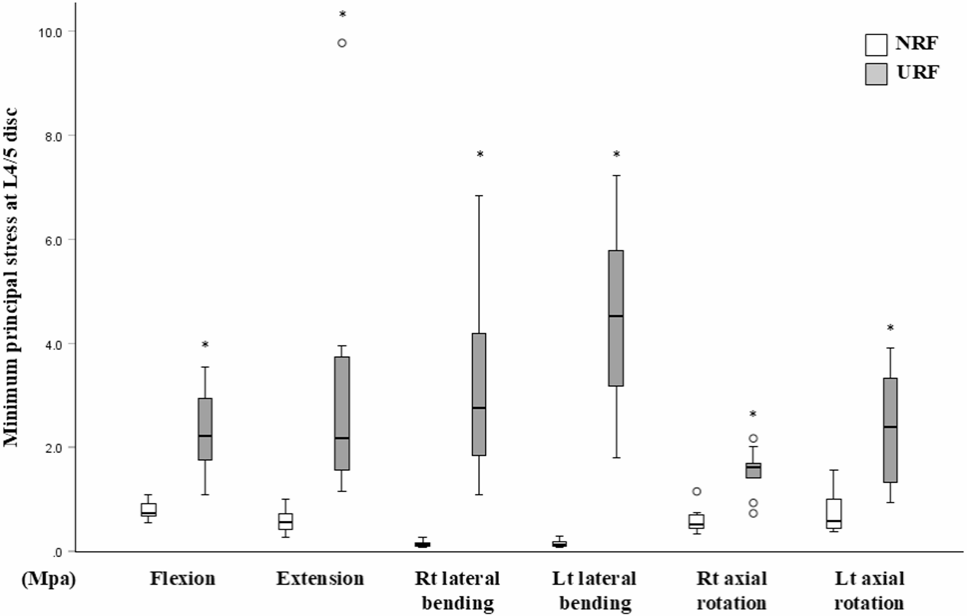
Minimum principal stress at L4/5 disc under 6 loading directions in the NRF (white box) and URF (grey box) models. A circle represents a mild outlier (more than 1.5 times the interquartile range beyond the box). * *p* < 0.05, versus. NRF model

At the adjacent L2/3 level, there was no significant difference in the minimum principal stress between the two groups under any loading direction (Fig. 2). However, at the level of rod fracture (L4/5), the minimum principal stress under all loading directions was significantly higher in the URF model versus the NRF model (Fig. 3). In short, the URF model demonstrated increased mechanical stress locally at the level of RF, but with minimum impact on the adjacent disc level.

### Mechanical stress on the rod

**Fig. 4:**
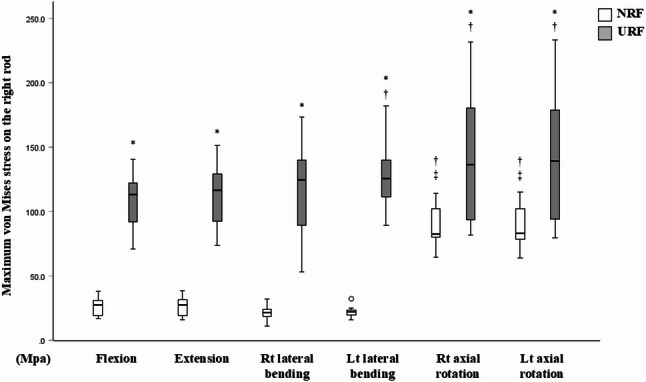
Maximum von Mises stress on the right rod under 6 loading directions in the NRF (white box) and URF (grey box) models. A circle represents a mild outlier (more than 1.5 times the interquartile range beyond the box). * *p* < 0.05, versus. NRF model; † *p* < 0.05, versus. flexion; ‡ *p* < 0.05, versus. extension and right/left lateral bending

**Fig. 5:**
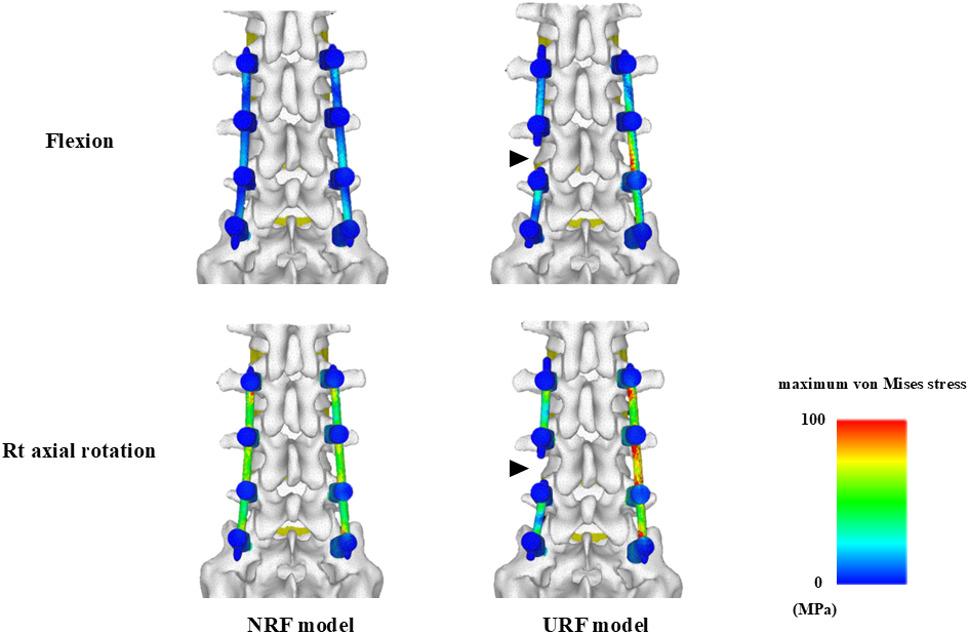
Contours of maximum von Mises stress on the rods under flexion (above) and right axial rotational (below) loadings in the NRF (left) and URF (right) models. Arrow head indicates RF site

The maximum von Mises stress on the right rod at L4/5 was significantly higher in the URF model under all loading directions (Fig. 4). In the NRF model, the maximum von Mises stress on the right rod at L4/5 was significantly greater under left and right axial rotational loadings versus the other loading directions (Fig. 4). In the URF model, the mechanical stress on the remaining right rod was significantly higher under left lateral bending and under both left and right axial rotational loading versus flexion (Fig. 4). Contours demonstrated increased mechanical stress along the entire non-screw-contact regions of the rod in the NRF model, as well as on the remaining right rod in the URF model, under right axial rotational loading versus flexion loading (Fig. 5). Thus, axial rotation induces substantial mechanical stress on the entire rod after posterior spinal fusion surgery. Meanwhile, axial rotation and lateral bending toward the fractured side of the rod increases mechanical stress on the remaining rod in the setting of URF.

## Discussion

The results of this study can be summarized into three main findings. First, URF significantly increases mechanical stress on the intervertebral disc at the level of RF, with minimal effect on the adjacent intervertebral disc. Second, URF substantially increases mechanical stress on the remaining rod under all loading conditions. Third, in both the NRF and URF models, axial rotational loading increased the mechanical stress on the rods, and in the URF model, lateral bending toward the fractured side further increased the mechanical stress. To our knowledge, this is the first study to analyze the change in mechanical stress on spinal rods under various loading directions using a URF model via CT/FEA. Our findings suggest that restricting axial rotation after spinal fusion surgery, and further limiting lateral bending toward the fractured side in cases of URF, may help reduce mechanical stress on the rods. From these biomechanical observations, postoperative motion instruction or bracing to limit rotation and lateral bending could potentially contribute to prevention of the occurrence or progression of RF. However, further biomechanical and clinical studies are needed to confirm these potential preventive effects for RF.

The increased stress at the level of RF (L4/5) in the URF model is expected, because the loss of unilateral rod continuity likely compromises mechanical stability at this level, thereby concentrating the mechanical stress across the intervertebral disc. Conversely, the absence of significant differences in mechanical stress at the adjacent level (L2/3) suggests that the biomechanical impact of URF may be relatively localized without substantially altering mechanical stress on the proximal segment, at least in short fusion model. Further investigation using a longer spinal fusion model is needed to evaluate the changes in mechanical stress at the adjacent level after URF.

In the NRF model, axial rotational loading increased mechanical stress across the entire non-screw-contact regions of rods. Meanwhile, in the URF model, the mechanical stress on the remaining rod significantly increased at the level of RF, and it further worsened under axial rotational loading and lateral bending loading toward the fractured side of the rod compared to flexion. This observed increase in the mechanical stress on the rods under axial rotational loading in both models suggests that reducing torsional stress on the rods may be important in preventing RF. In line with this, previous studies have emphasized the potential benefits of cross-links in reducing the torsional loads on rods [27–30]. Rotational forces are known to concentrate torsional stress on the rods, thereby potentially exceeding the material fatigue limits over time. The use of cross-links can mitigate this stress by stabilizing the construct against axial rotation, which can help prevent RF. Wang et al. analyzed a post-osteotomy spinal construct using finite element modeling, reporting that rotational loading produced the highest rod stress, but this could be alleviated by the placement of a cross-link [30]. However, that study was limited to a single case model, lacked direct comparisons across different loading directions, and did not evaluate this under a URF condition. In contrast, our study created finite element models based on the CT data of 10 different patients and assessed the mechanical stress on the rods under various loading directions using a novel URF model. As a result, our results suggest that restricting axial rotation after posterior spinal fusion surgery may help prevent the occurrence and progression of RF. Although surgical strategies (e.g., using stiffer rod materials, multiple-rod constructs, or adding cross-links) remain essential for increasing construct rigidity and dispersing rotational stress, our findings also highlight the importance of postoperative preventive strategies. To reduce mechanical stress on the rods, patients may benefit from guidance on limiting trunk rotation and from the possible use of a thoracolumbosacral orthosis to restrict spinal axial motion. Nevertheless, further prospective studies are warranted to investigate the actual clinical impact of postoperative bracing or spinal motion instruction in terms of preventing RF after spinal fusion surgery.

This study has several limitations. First, to minimize potential confounders such as spinal alignment and deformity, this study excluded patients with severe lumbar degenerative changes and kyphoscoliosis and adopted a simplified short-segment spinal fusion model (L3–S1) without interbody cage or bone graft. This approach limits the generalizability of our findings to the adult spinal deformity patients or actual spinal fusion surgery. Further investigation using a longer spinal fusion model with interbody cage or bone graft in patients with adult spinal deformity is needed. Second, there is a temporal discrepancy between clinical RF and our analysis. RF develops postoperatively as a time-dependent fatigue process, whereas our finite element analysis is static and based on preoperative geometry. Thus, our findings should be interpreted as showing baseline biomechanical insights rather than a complete representation of the clinical course. Third, this study did not include direct experimental or clinical validation of the finite element model against benchmark data. Fourth, the intervertebral discs were modeled as homogeneous structures using a Mooney-Rivlin hyperelastic model. As a result, the modeled discs may have exhibited higher stiffness, potentially contributing to the relatively large absolute values of minimum principal stress. Future studies using more anatomically detailed disc models may help provide a more physiologically accurate representation of mechanical stress on the intervertebral discs. Fifth, the bone quality and bone mineral density of the patients in this study were not evaluated, and the numbers of male and female patients differed (6 males and 4 females); therefore, these factors may have influenced the results. Sixth, this study did not perform a mesh convergence analysis to confirm the independence of the results from the mesh density. Although the mesh size was determined based on validated lumbar spine finite element models [20, 21], performing a mesh convergence analysis in future work would be important to further validate the model used in the present study. Seventh, in this study, the contact between the pedicle screw and the vertebrae was assumed to be perfectly bonded, which may affect the mechanical stress distribution on the rods. Finally, this study did not evaluate the mechanical stress on rods caused by nonunion, which is widely recognized as the primary cause of RF. Therefore, the clinical impact of our findings is limited. Nevertheless, by using finite element models under controlled settings, this study clarified that axial rotation substantially increases the mechanical stress on the rods in both NRF and URF models. These results provide complementary biomechanical insights into the mechanisms underlying the occurrence and progression of RF.

## Conclusions

This study clarified that axial rotation increases mechanical stress on spinal rods after posterior spinal fusion surgery, while axial rotation and lateral bending toward the fractured side of the rod increase mechanical stress on the remaining rod in the setting of URF. These results suggest that restricting axial rotation after posterior spinal fusion surgery, as well as restricting rotation and lateral bending toward the rod fractured side in cases of URF, may help reduce mechanical stress on the rods. While such motion restrictions could potentially contribute to preventing the occurrence or progression of RF, further biomechanical and clinical studies are needed to validate these implications.

## Data Availability

The datasets generated and/or analyzed during the current study are not publicly available due to their containing information that could compromise the privacy of research participants but are available from the corresponding author on reasonable request.

## Abbreviations

BMI: Body mass index
CT/FEA: 3D-computed tomography finite element analysis
IQR: Interquartile range
MPa: Megapascal
NRF: No rod fracture
RF: Rod fracture
SD: Standard deviation
URF: Unilateral rod fracture

## References

[1] Diebo BG, Shah NV, Boachie-Adjei O, Zhu F, Rothenfluh DA, Paulino CB, Schwab FJ, Lafage V. Adult spinal deformity. Lancet. 2019;394(10193):160–72. 31305254 10.1016/S0140-6736(19)31125-0

[2] Kebaish KM, Neubauer PR, Voros GD, Khoshnevisan MA, Skolasky RL. Scoliosis in adults aged forty years and older: prevalence and relationship to age, race, and gender. Spine (Phila Pa 1976). 2011;36(9):731–6. 20881515 10.1097/BRS.0b013e3181e9f120

[3] Kelly MP, Kallen MA, Shaffrey CI, Smith JS, Burton DC, Ames CP, Lafage V, Schwab FJ, Kim HJ, Klineberg EO, et al. Examining the patient-reported outcomes measurement information system versus the scoliosis research society-22r in adult spinal deformity. J Neurosurg Spine. 2019;30(6):801–6. 30797200 10.3171/2018.11.SPINE181014

[4] Schwab F, Dubey A, Gamez L, El Fegoun AB, Hwang K, Pagala M, Farcy JP. Adult scoliosis: prevalence, SF-36, and nutritional parameters in an elderly volunteer population. Spine (Phila Pa 1976). 2005;30(9):1082–5. 15864163 10.1097/01.brs.0000160842.43482.cd

[5] Kim HJ, Yang JH, Chang DG, Lenke LG, Suh SW, Nam Y, Park SC, Suk SI. Adult spinal deformity: a comprehensive review of current advances and future directions. Asian Spine J. 2022;16(5):776–88. 36274246 10.31616/asj.2022.0376 PMC9633249

[6] Soroceanu A, Diebo BG, Burton D, Smith JS, Deviren V, Shaffrey C, Kim HJ, Mundis G, Ames C, Errico T, et al. Radiographical and implant-related complications in adult spinal deformity surgery: incidence, patient risk factors, and impact on health-related quality of life. Spine (Phila Pa 1976). 2015;40(18):1414–21. 26426712 10.1097/BRS.0000000000001020

[7] Burke JF, Scheer JK, Lau D, Safaee MM, Lui A, Jha S, Jedwood C, Thapar I, Belfield B, Nobahar N, et al. Failure in Adult Spinal Deformity Surgery: A Comprehensive Review of Current Rates, Mechanisms, and Prevention Strategies. Spine (Phila Pa 1976). 2022;47(19):1337–50. 36094109 10.1097/BRS.0000000000004435

[8] Sardi JP, Lazaro B, Smith JS, Kelly MP, Dial B, Hills J, Yanik EL, Gupta M, Baldus CR, Yen CP, et al. Rod fractures in thoracolumbar fusions to the sacrum/pelvis for adult symptomatic lumbar scoliosis: long-term follow-up of a prospective, multicenter cohort of 160 patients. J Neurosurg Spine. 2023;38(2):217–29. 36461845 10.3171/2022.8.SPINE22423 PMC10193478

[9] Smith JS, Shaffrey CI, Ames CP, Demakakos J, Fu KM, Keshavarzi S, Li CM, Deviren V, Schwab FJ, Lafage V, et al. Assessment of symptomatic rod fracture after posterior instrumented fusion for adult spinal deformity. Neurosurgery. 2012;71(4):862–7. 22989960 10.1227/NEU.0b013e3182672aab

[10] Uribe JS, Deukmedjian AR, Mummaneni PV, Fu KM, Mundis GM Jr., Okonkwo DO, Kanter AS, Eastlack R, Wang MY, Anand N, et al. Complications in adult spinal deformity surgery: an analysis of minimally invasive, hybrid, and open surgical techniques. Neurosurg Focus. 2014;36(5):E15. 10.3171/2014.3.FOCUS13534 24785480

[11] Lertudomphonwanit T, Kelly MP, Bridwell KH, Lenke LG, McAnany SJ, Punyarat P, Bryan TP, Buchowski JM, Zebala LP, Sides BA, et al. Rod fracture in adult spinal deformity surgery fused to the sacrum: prevalence, risk factors, and impact on health-related quality of life in 526 patients. Spine J. 2018;18(9):1612–24. 29501749 10.1016/j.spinee.2018.02.008

[12] Glassman SD, Anagnost SC, Parker A, Burke D, Johnson JR, Dimar JR. The effect of cigarette smoking and smoking cessation on spinal fusion. Spine (Phila Pa 1976). 2000;25(20):2608–15. 11034645 10.1097/00007632-200010150-00011

[13] Govindarajan V, Diaz A, Perez-Roman RJ, Burks SS, Wang MY, Levi AD. Osteoporosis treatment in patients undergoing spinal fusion: a systematic review and meta-analysis. Neurosurg Focus. 2021;50(6):E9. 10.3171/2021.3.FOCUS2175 34062507

[14] Jackson KL 2nd, Devine JG. The effects of obesity on spine surgery: a systematic review of the literature. Global Spine J. 2016;6(4):394–400. 27190743 10.1055/s-0035-1570750 PMC4868585

[15] Khalid SI, Nunna RS, Maasarani S, Belmont E, Deme P, Chilakapati S, Eldridge C, Singh R, Bagley CA, Adogwa O. Association of osteopenia and osteoporosis with higher rates of pseudarthrosis and revision surgery in adult patients undergoing single-level lumbar fusion. Neurosurg Focus. 2020;49(2):E6. 10.3171/2020.5.FOCUS20289 32738806

[16] Smith JS, Shaffrey E, Klineberg E, Shaffrey CI, Lafage V, Schwab FJ, Protopsaltis T, Scheer JK, Mundis GM Jr., Fu KM, et al. Prospective multicenter assessment of risk factors for rod fracture following surgery for adult spinal deformity. J Neurosurg Spine. 2014;21(6):994–1003. 25325175 10.3171/2014.9.SPINE131176

[17] Lee KY, Lee JH, Kang KC, Im SK, Lim HS, Choi SW. Strategies for prevention of rod fracture in adult spinal deformity: cobalt chrome rod, accessory rod technique, and lateral lumbar interbody fusion. J Neurosurg Spine. 2021;34(5):706–15. 33607617 10.3171/2020.8.SPINE201037

[18] Kozaki T, Tsutsui S, Yamamoto E, Murata A, Nakanishi R, Yamada H. Quantitative biomechanical evaluation for optimal spinal instrumentation to prevent mechanical complications in spinal fusion from the lower thoracic spine to the pelvis for adult spinal deformity: a finite element Analysis. Spine Surg Relat Res. 2023;7(3):276–83. 37309490 10.22603/ssrr.2022-0131 PMC10257959

[19] Leszczynski A, Meyer F, Charles YP, Deck C, Bourdet N, Willinger R. Influence of double rods and interbody cages on range of motion and rod stress after spinopelvic instrumentation: a finite element study. Eur Spine J. 2022;31(6):1515–24. 35461384 10.1007/s00586-022-07149-3

[20] Kozaki T, Hashizume H, Oka H, Ohashi S, Kumano Y, Yamamoto E, Minamide A, Yukawa Y, Iwasaki H, Tsutsui S, et al. Lumbar fusion including sacroiliac joint fixation increases the stress and angular motion at the hip joint: a finite element study. Spine Surg Relat Res. 2022;6(6):681–8. 36561150 10.22603/ssrr.2021-0231 PMC9747219

[21] Kozaki T, Lundberg HJ, Mell SP, Samartzis D, Kawakami M, Yamada H, Inoue N, An HS. Effect of lumbar fusion and pelvic fixation rigidity on hip joint stress: a finite element analysis. Spine (Phila Pa 1976). 2023;48(20):E355–61. 37530119 10.1097/BRS.0000000000004791

[22] Bathe KJ, Finite Element Procedures. 2006

[23] Keyak JH, Rossi SA, Jones KA, Skinner HB. Prediction of femoral fracture load using automated finite element modeling. J Biomech. 1998;31(2):125–33. 9593205 10.1016/s0021-9290(97)00123-1

[24] Kim HJ, Chun HJ, Kang KT, Lee HM, Kim HS, Moon ES, Park JO, Hwang BH, Son JH, Moon SH. A validated finite element analysis of nerve root stress in degenerative lumbar scoliosis. Med Biol Eng Comput. 2009;47(6):599–605. 19296142 10.1007/s11517-009-0463-y

[25] Natarajan RN, Andersson GB. The influence of lumbar disc height and cross-sectional area on the mechanical response of the disc to physiologic loading. Spine (Phila Pa 1976). 1999;24(18):1873–81. 10515010 10.1097/00007632-199909150-00003

[26] Panjabi MM, White AA, 3rd. Basic biomechanics of the spine. Neurosurgery. 1980;7(1):76–93. 7413053 10.1227/00006123-198007000-00014

[27] Alizadeh M, Kadir MR, Fadhli MM, Fallahiarezoodar A, Azmi B, Murali MR, Kamarul T. The use of X-shaped cross-link in posterior spinal constructs improves stability in thoracolumbar burst fracture: a finite element analysis. J Orthop Res. 2013;31(9):1447–54. 23640802 10.1002/jor.22376

[28] Brodke DS, Bachus KN, Mohr RA, Nguyen BK. Segmental pedicle screw fixation or cross-links in multilevel lumbar constructs. a biomechanical analysis. Spine J. 2001;1(5):373–9. 14588318 10.1016/s1529-9430(01)00116-4

[29] Lehman RA Jr., Kang DG, Wagner SC, Paik H, Cardoso MJ, Bernstock JD, Dmitriev AE. Biomechanical stability of transverse connectors in the setting of a thoracic pedicle subtraction osteotomy. Spine J. 2015;15(7):1629–35. 25771755 10.1016/j.spinee.2015.03.010

[30] Wang T, Cai Z, Zhao Y, Wang W, Zheng G, Wang Z, Wang Y. The influence of cross-links on long-segment instrumentation following spinal osteotomy: a finite element analysis. World Neurosurg. 2019;123:e294–302. 30496922 10.1016/j.wneu.2018.11.154

